# Meiosis in Polyploids and Implications for Genetic Mapping: A Review

**DOI:** 10.3390/genes12101517

**Published:** 2021-09-27

**Authors:** Nina Reis Soares, Marcelo Mollinari, Gleicy K. Oliveira, Guilherme S. Pereira, Maria Lucia Carneiro Vieira

**Affiliations:** 1 Escola Superior de Agricultura “Luiz de Queiroz”, Universidade de São Paulo, Piracicaba 13400-918, Brazil; ninareissoares@usp.br (N.R.S.); gleicy.k.oliveira@gmail.com (G.K.O.); g.pereira@ufv.br (G.S.P.); 2 Bioinformatics Research Center, North Carolina State University, Raleigh, NC 27695-7566, USA; mmollin@ncsu.edu; 3 Department of Horticultural Science, North Carolina State University, Raleigh, NC 27695-7555, USA; 4 Department of Agronomy, Federal University of Viçosa, Viçosa 36570-900, Brazil

**Keywords:** auto- and allopolyploids, meiosis, early meiosis, genetic control, homoeologous recombination, genetic maps, allelic dosage, multilocus linkage

## Abstract

Plant cytogenetic studies have provided essential knowledge on chromosome behavior during meiosis, contributing to our understanding of this complex process. In this review, we describe in detail the meiotic process in auto- and allopolyploids from the onset of prophase I through pairing, recombination, and bivalent formation, highlighting recent findings on the genetic control and mode of action of specific proteins that lead to diploid-like meiosis behavior in polyploid species. During the meiosis of newly formed polyploids, related chromosomes (homologous in autopolyploids; homologous and homoeologous in allopolyploids) can combine in complex structures called multivalents. These structures occur when multiple chromosomes simultaneously pair, synapse, and recombine. We discuss the effectiveness of crossover frequency in preventing multivalent formation and favoring regular meiosis. Homoeologous recombination in particular can generate new gene (locus) combinations and phenotypes, but it may destabilize the karyotype and lead to aberrant meiotic behavior, reducing fertility. In crop species, understanding the factors that control pairing and recombination has the potential to provide plant breeders with resources to make fuller use of available chromosome variations in number and structure. We focused on wheat and oilseed rape, since there is an abundance of elucidating studies on this subject, including the molecular characterization of the *Ph1* (wheat) and *PrBn* (oilseed rape) loci, which are known to play a crucial role in regulating meiosis. Finally, we exploited the consequences of chromosome pairing and recombination for genetic map construction in polyploids, highlighting two case studies of complex genomes: (i) modern sugarcane, which has a man-made genome harboring two subgenomes with some recombinant chromosomes; and (ii) hexaploid sweet potato, a naturally occurring polyploid. The recent inclusion of allelic dosage information has improved linkage estimation in polyploids, allowing multilocus genetic maps to be constructed.

## 1. Overview

The study of meiosis in polyploid species began in the 1920s with the classic report of Newton and Darlington (1929) [1], who studied triploid and pentaploid tulips. A number of important studies on meiotic variations in polyploids have been published over the past century, particularly in the last few decades [2,3,4,5,6,7,8,9,10]. There are two classes of naturally occurring polyploids: autopolyploids, which have three or more copies of the same genome (e.g., the autotetraploid *Solanum tuberosum*, 2*n* = 4*x* = 48), and allopolyploids, which are the result of interspecific hybridization between related progenitors and genome doubling (e.g., the allotetraploid *Nicotiana tabacum*, 2*n* = 4*x* =24, whose genome composition is AABB). However, this classification is relatively flexible. For example, chromosome sets in allopolyploids differ in proportion to the divergence level between the parental genomes: the closer the parents, the more similar the resulting allopolyploid is to an autopolyploid [11]. During the meiosis of newly formed polyploids, related chromosomes (homologous in autopolyploids; homologous and homoeologous in allopolyploids) may pair and combine in complex structures called multivalents (autopolyploids) or form illegitimate homoelogous pairing (allopolyploids) [12,13,14,15]. These structures occur when multiple chromosomes simultaneously pair, synapse, and recombine. Multivalents or homeologous pairings that reach metaphase I (MI) are related to segregation issues, leading to aneuploid gametes, compromised fertility, and low fitness of offspring [16].

## 2. Revisiting Early Meiosis

**Figure 1:**
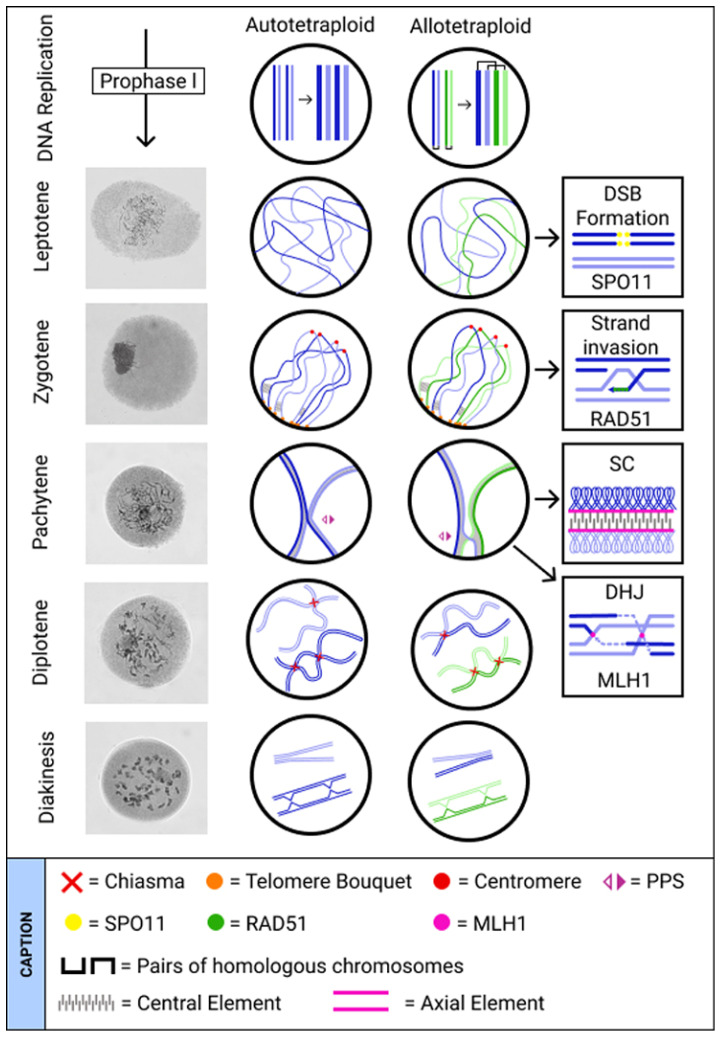
Pre-meiotic and early meiotic events in auto- and allotetraploid species illustrating regular meiotic behavior during Prophase I. Meiosis is preceded by one round of DNA replication during which sister chromatids are duplicated. During leptotene, genetic recombination is initiated, and double-strand breaks (DSBs) are catalyzed by the protein Spo11 and repaired through homologous recombination. This process leads to DSB invasion into the non-sister chromatid by the RAD51 protein, initiating physical interactions and driving chromosome sorting [30,31,32]. Homologous recognition may be facilitated by the clustering of telomeres at one pole of the cell, forming the telomere bouquet (see [156]). During zygotene, chromosomes begin synapsis via the formation of the synaptonemal complex (SC), which consists of axial and central elements [33]. The SC is formed between pairs of homologous chromosomes, but it can also be formed between more than two homologs in autotetraploids and between homoeologous chromosomes in allotetraploids, resulting in synaptic partner switches (PPS) at pachytene. As recombination proceeds, in some species, multiple/homoeologous associations are corrected by the MLH1 protein, which is a DNA mismatch repair that is required to resolve DHJ into COs. By contrast, in other species, the specific localization of crossovers between pairs of homologous chromosomes resolves multiple⁄homoeologous associations at diplotene, when the SC is disassembled (see [150]). Irrespective of when the corrections occur, only bivalents are visualized at diakinesis when chromosomes recondense in established polyploid lineages. Modified from Cifuentes et al. (2010) [5]. On the left, prophase I images depict a commercial variety of sugarcane. Photo credit: Oliveira, G.K., Universidade de São Paulo, Brazil.

The premeiotic organization of homologous chromosomes in polyploid and diploid species is very similar. Homologous chromosomes are nonrandomly distributed and organized in the nucleus (Figure 1) [17,18]. In many eukaryotes, telomeres and centromeres cluster at opposite poles of the nucleus during prophase I (PI) [19], forming the Rabl-configuration, first described by the pioneering studies of Rabl in 1885 and Bovery in 1909 see [20]. In *Arabidopsis*, in which a non-Rabl pattern of chromosome organization occurs, dominant ‘chromosome territories’ arise, consisting of heterochromatic centromeric regions at the nuclear periphery, from which chromosome arms emanate [21]. In this case, the chromosome position is also associated with the gene expression level [22,23].

Then, chromosomes must move at the beginning of meiosis to find the correspondent homolog. This movement can be telomere oriented to form the bouquet or involve chromatin unfolding [24,25]. Once the chromosomes are close enough to interact, potential partners must be chosen. This process is mediated by homology. A certain degree of sequence identity is required, and pairing depends on the type of polyploidy level, species, individual chromosomes, and chromosome segments (Figure 1) [26,27].

Early stages of recognition and pairing can be more complex in polyploids than in diploids; this is caused by the high number of potential homologous partners that can delay the progression of meiosis [28]. During the telomere bouquet phase in early meiosis, centromere associations begin the process of sorting chromosomes. When more than one complement of chromosomes is present, early association of the centromeres is triggered in auto- and allopolyploids [29], leading to the formation of multicentromeric clusters, which help resolve nonhomologous centromere associations, thereby contributing to homologous chromosome sorting (Figure 1) [29].

After chromosomes are associated by their centromeres, the process of homologous chromosome alignment begins. First, the programmed double-strand DNA breaks (DSBs) produced in early leptotene are catalyzed by the topoisomerase-like protein, Spo11, causing physical interactions, which lead to chromosome sorting [30,31]. After a DSB occurs, each chromosome has two or more potential partners to interact with in order to repair the DSBs by homologous recombination using a non-sister chromatid as a template. Fragments of DNA around the 5′-end of the break are nicked during resection, and the overhanging 3′-end of the broken DNA molecule associates with recombinase RAD51 and/or the meiosis-specific recombinase DMC1, which invades a similar or identical DNA molecule: a nucleoprotein filament [32]. Shortly after the chromosomes align, they are united by the synaptonemal complex (SC), which is a stable proteinaceous structure [28]. The SC is formed by three elements: the axial element (AE), central element (CE), and recombination nodules (RNs) (Figure 1) [33]. The RNs are complexes of several proteins involved in synapsis and recombination [34]. After the assembly of the SC in zygotene, homologous chromosomes become fully synapsed at pachytene [35,36]. Homologous chromosomes are kept together along their length by the SC, which also serves as a scaffold to recruit factors of the recombinational repairing machinery [37].

Interestingly, during PI, a range of SC assembly variations may occur, including multiple SCs and synaptic multivalents exhibiting pairing partner switches (PPS). The most frequent meiotic variation in polyploids is the occurrence of complex synaptic interactions, when the progressive pairing of three or more chromosomes starts simultaneously at different points along their lengths, producing PPS [2]. PPS distribution is irregular but not accidental (Figure 1). The existence of more than one switch per chromosome implies the presence of additional autonomous pairing sites (APS) along the chromosomes, each with a low probability of generating a PPS [38]. Pairing with one chromosome at one APS promotes the continuation of pairing in a zipper-like manner [28]. Variations in meiosis are mainly due to the type of presynaptic alignment, either distal or complete; the number and distribution of synapsis initiation points; the number of partner exchanges and progress through zygotene and pachytene; and whether or not there are preferences in partner selection [39]. These synaptic multivalents can be observed in auto- and allopolyploid plants, with PPS occurring more often in triploids than in tetraploids [6,38,40,41].

## 3. Meiosis in Autopolyploids

Autopolyploidy occurs in individuals or species that have undergone a whole genome duplication (WGD) event, due to non-disjunction of the gametes during meiosis, resulting in 2*n* gametes rather than haploid (*n*) gametes. When these gametes are fertilized, they can produce triploid (2*n* + *n*) or tetraploid (2*n* + 2*n*) individuals. Therefore, an autopolyploid has more than two copies of homologous chromosomes that are equally capable of randomly pairing, synapsing and recombining (crossing over) during PI. When these events are observed in more than two homologous chromosomes, a multivalent can be formed at MI, and chromosome missegregation can occur at anaphase I (AI) [27] (Figure 1). On the one hand, most of these multivalents are dissolved prior to MI in established autopolyploids, which primarily form bivalents. On the other hand, multivalents are frequently retained in resynthesized autopolyploids, mainly as tri- and/or tetravalents (or quadrivalents). The occurrence of multivalents depends on the ploidy level and homology between chromosomes [42], and it is known to be controlled by genetic factors [7,9]. Metaphase I multivalents are associated with an increased risk of homologous missegregation at AI. However, molecular mechanisms have evolved to reduce the meiotic challenges faced by polyploids, generating fertile autopolyploids [7,8,9].

## 4. Frequency of Crossovers

In contrast to populations of natural, well-established autopolyploids, natural neo- autopolyploids often have a high number of multivalents at MI resulting in high levels of aneuploidy and consequently in low fertility [43,44]. When an autopolyploid is resynthesized and selected for meiotic stabilization, resulting therefore in successful chromosome transmission, fewer multivalents are observed, which is followed by a reduction in the number of crossovers (CO) [9]. In plants, as the number of initial DSBs exceeds the number of COs, the majority of DSBs are resolved, but a minority fraction (≈5%) results in CO [45,46,47,48,49]. When chromosomes form COs with more than one partner, multivalents are observed at MI. Therefore, ensuring that each chromosome has only one CO prevents the formation of multivalents. In many natural species, decreased CO frequencies have been observed in evolved tetraploids compared to their diploid precursor [13,50,51,52].

Notably, there are no correlations between chromosome length and the number of COs [53,54]. In fact, genomes consist of ‘hot and cold spots’, with respective high and low rates of meiotic recombination [54,55,56], suggesting that reduced CO frequency is an effective path toward meiotic adaptation. Results reported in *Arabidopsis* support the idea that one CO per chromosome is associated with low multivalent frequency in the natural autotetraploid *Arabidopsis arenosa*, evidencing the effect of genes on CO rates [13,50]. In *A. thaliana*, the CO number increases in newly formed polyploids [44], with fewer multivalents seen after a few generations [57,58]. Thus, while there may be a temporary increase in COs, it seems that evolution favors reductions in CO rates in the longer term, at least in autotetraploids.

CO positioning ensures that every chromosome copy has at least one CO, thus excluding ‘zero-CO’ univalents and trivalent-plus-univalent configurations [7]. Some insights into which genes might be involved in this process emerged from a comparison between diploid and tetraploid *A. arenosa* genomes, identifying several meiotic genes as targets for selection in the tetraploid lineage, where most of these genes encode chromosome axis components such as Asy1, Asy3, and Syn1/ Rec8, or their direct interactors Zip1a/Zip1b and Pds5 [13,59].

## 5. Multivalents and Cytological Diploidization

Polyploid meiotic configurations during MI may comprise the presence of univalents, bivalents, and multivalents. In 1947, Stebbins [60] had already pointed out that multivalents are disadvantageous due to their negative impact on fertility and karyotype stability. Several subsequent studies support this view, determining that selection for fertility results in fewer multivalents and produces a cytological diploidization, in which chromosomes predominantly (or exclusively) pair as bivalents in MI [43,57]. 

Nevertheless, studies on autotetraploids show that high fertility correlates with increased quadrivalents at MI [61,62], suggesting that univalents and trivalents are the main cause of reduced fertility, probably because they are less likely to segregate appropriately. In established autotetraploid lines of *A. thaliana* (ecotype Columbia), fewer multivalents were observed than in newly synthesized lines, suggesting that partial cytological diploidization occurred over 13 generations [57]. 

A reduction in the number of multivalents has been observed through PI and from PI to MI in polyploids, regardless of the presence of univalents [5]. Some multivalents formed during zygotene are resolved into bivalents in pachytene, due either to the removal of SC sections or to the suppression of recombination so that only bivalent associations are retained [37,62]. The correction of multivalent associations continues during late zygotene. However, a proportion of multivalents persists in most cases. Overall, the process of diploidization in meiotic behavior is a common strategy throughout the evolution of autopolyploid species [49,62,63,64,65,66].

Autopolyploids also exhibit idiosyncrasies in allelic segregation. While both diploids and allopolyploids display disomic inheritance, autopolyploids exhibit polysomic inheritance. Polysomic inheritance is the result of (i) the random assortment of multiple homologous chromosomes; (ii) a series of dosage allelic combinations (for instance, AAAA, AAAa, AAaa, Aaaa, and aaaa for an autotetraploid); and (iii) the presence of more than two alleles at a locus. Such polysomic inheritance occurs in autopolyploids irrespective of the presence of multivalents or bivalents at MI, which is a point that will be addressed later. Disomic and polysomic inheritance are only extreme cases on a gradient, with intermediate inheritance patterns taking place when every chromosome has a preferential, but not exclusive, partner [67]. In contrast, multivalent formation is often associated with ‘double reduction’, when distal segments of sister chromatids end up in the same gamete, i.e., in one gamete two copies of the same gene sequence (allele) will be derived from the same parental chromosome [27]. All these attributes directly impact population genetic parameters with possible consequences for evolution [68]. In addition, such attributes might significantly influence genetic mapping, which is especially relevant for crop species. 

## 6. Genetic Control of Meiosis in Autopolyploids

In autopolyploids, only a few studies have identified genes regulating chromosomal pairing, synapsis, and CO occurrence. In the autotetraploid *A. arenosa*, some meiotic genes (Asy1, Asy3, Pds5b, Prd3, Rec8, Smc3, Zyp1a, and Zyp1b) encode proteins that coordinate early meiotic functions [13]. In early PI, the chromosomal axis present in each of the sister chromatids is transformed into two SC lateral elements, which are composed of a scaffold of cohesin proteins (SMC1, SMC3, PDS5, REC8, and SCC3) [69,70,71,72,73]. These proteins arrange the two sister chromatids into loops that are turned away from the axis (Figure 1) [74]. The tethered loop axis model proposes that meiotic DSBs are generated on the chromatin loops that become connected to the axis during inter-homolog repair [4,75]. ASY1, ASY3, and ASY4 are meiosis-specific proteins that end up in the cohesin scaffold, increasing inter-homolog recombination [76,77,78]. Furthermore, ASY1 and ASY3 are essential for establishing precise chromosomal pairing and synapsis [77,79,80]. 

Morgan et al. (2020) [81] investigated the effects of the aforementioned genes Asy1 and Asy3 on the autotetraploid *A. arenosa*, focusing on the derived (autotetraploid, T) and ancestral (diploid, D) alleles. The ASY1 and ASY3 proteins constitute the chromosome axis. These protein structures are formed along the replicated chromosomes during PI and are necessary for chromosome pairing, synapsis, and homologous recombination [4]. As a result, mutants for Asy1 or Asy3 are found to be deficient in synapsis and have low levels of COs and high levels of univalents [77,82]. The presence of “rod-shaped” bivalents, a shorter chromosome axis, and a reduction in multivalent association are all associated with the derived alleles Asy1 and Asy3 [81].

Derived alleles of Asy1 and, to a lesser extent, Asy3, are linked to a higher frequency of bivalents at MI with “rod-like” shapes, which are thought to indicate more distant CO locations [83]. Asy1 and Asy*3* mutants in *A. thaliana* contain relatively few COs, but those that do exist are mostly subtelomeric [79,83]. Centromeres and surrounding repetitive sequences (pericentromeric heterochromatin) are frequently suppressed for meiotic recombination; therefore, high CO levels are typically observed at distal subtelomeric regions that also tend to have higher gene density [84,85,86].

The derived alleles (T or D) of Asy1 and Asy3 may be responsible for the reduced CO number in autotetraploid *A. arenosa*, preventing multivalent formation by reducing the CO number to one per bivalent, ensuring that only bivalents are formed [7]. Plants homozygous for Asy1-T alleles exhibited less multivalent cells at MI than Asy1-D homozygotes, suggesting that Asy1-T alleles are important for meiotic stability. This is supported by the fact that Asy1-TTTT plants exhibit fewer synaptic partner switches and reduced multivalent formation in PI. In the presence of the Asy1-T allele, the reduction in multivalent formation may be determined, at least in part, by a higher propensity for COs to be positioned on the same side as partner switch sites [7]. The fact that Asy1 allele status may affect the axis length supports the idea that allelic variation at Asy1 might change axis organization in some way, and it is possible that this encourages the “safer” placement of CO sites on the same side as partner switch sites.

However, the factors and mechanisms that shape the meiotic recombination landscape along chromosomes remain to be understood. This could be related to the fact that telomeres cluster in PI, and this clustering is largely preserved in the *A. thaliana asy1* mutant, possibly allowing interhomolog recombination events to proceed in these regions due to the close proximity of the chromosomes [50,87]. Lambing et al. (2020) [88], using chromatin immunoprecipitation, reported an ascending gradient of the protein ASY1 from telomeres to centromeres, and this differential distribution along the arms is required for more equally distributing recombination. However, despite the concentration of ASY1 in centromeric areas, meiotic DSBs and COs are repressed due to the high concentration of heterochromatin in these areas [84,85,89].

In order to further study Asy3 gene influence in autopolyploids, Seear et al. (2020) [90] traced the evolutionary origins of the autotetraploid lines of *A. lyrata* and *A. lyrata* x *A. arenosa* hybrid populations, demonstrating that Asy3 stabilizes autotetraploid male meiosis. A novel allele was discovered, harboring a tandem duplication (TD) in a serine-rich region of the ASY3 protein, which is correlated with stable meiotic phenotypes in tetraploids.

In comparison to diploid *A. lyrata*, the number of COs in autotetraploid individuals dropped, with a reduction of COs in proximal and interstitial areas, thus indicating a basic procedure for meiotic adaptation to autopolyploidy [90,91]. Therefore, the ASY3 TD protein, present in autopolyploids, may be hypomorphic and act in distancing COs [77,78,91].

The Asy3 TD allele possibly arose from diploid *A. lyrata*, according to a phylogenetic study of the Asy3 alleles [92]. Bidirectional gene flow among *A. arenosa* and *A. lyrata* tetraploids increased the gene pool from which favorable alleles may be acquired and chosen, and new gene conversion chimeric alleles can accurately combine favorable sequences from different origins to enhance adaptation. The genesis of the adaptive Asy3 TD allele in tetraploid populations appears to be recent, although it is widely disseminated and introgressed within strict bounds in the *A. lyrata* × *A. arenosa* hybrid genomes studied [92]. Gene flow of adaptive alleles (Asy1, Prd3, Rec8, Smc3, Zyp1a, and Zyp1b) from *A. arenosa* may have been necessary to establish meiotic stability in recent *A. lyrata* tetraploids prior to the origin of the Asy3 TD allele, but this requirement has since been relaxed due to the presence of the dominant Asy3 TD allele. Additionally, gene flow has introduced Asy1, Prd3, Rec8, Smc3, Zyp1a, and Zyp1b alleles from *A. arenosa* into tetraploid *A. lyrata* [90].

## 7. Meiosis in Allopolyploids

Allopolyploids are formed by the fusion of unreduced gametes followed by genome doubling in F_1_ hybrids or interspecific or intergeneric hybridization [10,11]. Therefore, allopolyploids carry two (or more) full complements of chromosomes, each from a distinct progenitor genome, thus forming homoeologous subgenomes, which are differentiated based on variations in chromosome architecture, DNA sequences, and gene order. Nevertheless, chromosomes retain some degree of genetic affinity and thus share genomic synteny. This genetic affinity allows homoeologs to compete with homologs during interactions such as recognition, alignment, SC assembly, and CO [39]. However, exclusive bivalent pairing at MI is essential to ensure regular homologous segregation at AI and consequently reproductive stability. Such diploid-like behavior is a result of genetic regulatory systems, as evidenced in wheat, *Avena sativa*, *Festuca arundinacea*, cotton, and and *Brassica napus* (reviewed in [93]).

During allopolyploid meiosis, chromosomes recombine with their closest related homolog, forming bivalents at diakinesis [44,94,95]. Bivalent formation is accomplished by two complementary systems: in one system, differences between homoeologs lead to preferential pairing between homologs; in the other, a genetic control can differentiate sets of chromosomes and prevent pairing between homeologs [96].

The classic explanation for complete cytogenetic diploidization holds that the absence of homology between chromosomes from different subgenomes prevents their association during the early stages of meiosis, resulting in homologous pairing (see [97]). Then, these chromosomes are associated as bivalents by chiasma formation prior to MI. In addition, the preference for homologs rather than homoeologs in allopolyploids also seems to be under genetic control. The most important studied example is the *Pairing homoeologous 1* (*Ph1*) locus in wheat [94]. 

In allopolyploids, diploidization can be accomplished by combining different processes during the interphase and early stages of meiosis [98,99]. It has been proposed that polyploid chromosome ordering starts with centromere association [29]. This is followed by the restriction of synapsis initiation between homoeologs, so that most of the paring at zygotene is between homologous chromosomes. In a third stage, the dissolution of SCs in homoeologs occurs before COs at pachytene, decreasing the frequency of multivalents. However, in most species, such a correction is insufficient, and some multivalents persist. Finally, multivalents are fully resolved in the last recombination stages, when the prevention of COs between synapsed homoeologous segments results exclusively in homologous bivalents (Figure 1) [93].

The visualization of chromosomes during interphase allows chromosome pairing to be examined before the first division. The initial reports on this subject indicated that centromeres associate in hexaploid wheat [100] and that a high level of homologous pairing is achieved upon entry into meiosis [101]. During early meiotic PI, telomeres aggregate on the nuclear envelope, forming a cluster or bouquet [102,103,104], facilitating homologous chromosome sorting. The timing of telomere bouquet formation differs across species, appearing earlier at PI in wheat and rye and later in maize [24,105,106]. After the formation of the telomere bouquet, SC formation is initiated near the telomeres during early PI, progressing lengthwise pairing as PI proceeds (Figure 1) [107,108,109]. 

As in autopolyploids, multiple homologs and homoeologs in allopolyploids align in early PI, forming multivalents at zygotene [95,107,110]. This results in pairing configurations such as cross-structures, rings, and chains during metaphase [111,112]. However, such pairing configurations occur less often in allopolyploids and are frequently restricted to smaller chromosome regions. During zygotene, these presynaptic associations may progress into synaptic partner switches [95,98,113] although to a lesser extent than in autopolyploids (Figure 1). 

In allopolyploids, recombination nodules are found in homoeologous synapsis regions [113], indicating that strand invasion and CO formation occur between homoeologous chromosomes. However, they do not progress as true COs [99]. At the time cells enter pachytene, the number of synaptic partner switches declines, and almost all chromosomes show homologous synapsis by the end of pachytene [95,114]. However, when compared to diploids, the number of COs per chromosome can be higher in allopolyploids, which present multiple sets of homoeologous chromosomes, as demonstrated in *Arabidopsis* [44], *Gossypium* [115], *Zea* [116], and *Brassica* [117,118]. For example, the genetic mapping of *Brassica napus* allotetraploids (AACC, 2*n* = 4*x* = 38), generated from the natural hybridization between *B. rapa* (AA, 2*n* = 2*x* = 20) and *B. oleracea* (CC, 2*n* = 2*x* = 18) [119], revealed around twice as many COs between the homologous A07 chromosomes than in the diploid AA hybrids [117]. A rise in the number of COs was also linked with a decrease in the CO interference strength [120]. The molecular mechanisms underlying this increase are unknown, but they appear to be dependent on the addition of specific C chromosomes, as demonstrated by Suay et al. (2014) [120], who demonstrated a non-additive dosage effect. Nevertheless, in allotriploid AAC *Brassica* hybrids, researchers observed a higher number of CO and the reshaping of the recombination landscapes when compared to diploid AA [118]. In allotriploids, the presence of the nine additional C chromosome leads to an increase in COs between all homologous A chromosomes, especially in the centromeres’ proximity, with a strong decrease in interference of Class I COs compared to the diploid AA [118].

In naturally occurring allopolyploids, homoeologous pairing is corrected by diplotene. Consequently, allopolyploids show homologous bivalent formations at MI and disomic inheritance [44,121]. The dissociation of chromosomes from multivalent configurations often leads to deficient gametes, with duplication and aneuploidy, ultimately resulting in reduced fertility [2]. Recombination between homoeologous chromosomes can be problematic: it contributes to the homogenization of the subgenomes, promoting further recombination between homoeologs, and it can eliminate the contribution of one parent in a genomic region, leading to gene dosage imbalance and other problems related to aneuploidy [122,123].

Importantly, recombination between homoeologs in established allopolyploids appears to be an uncommon phenomenon. Therefore, fertile allopolyploids either had some level of pre-existing control over pairing or must have acquired such genetic control during their evolution. It is also possible that structural changes occurring in newly formed polyploids (e.g., expansion or contraction of repeat elements or other genomic rearrangements) contributed to divergence among homoeologs and facilitated correct homolog pairing [122].

## 8. Homoeologous Exchanges

In many allopolyploid species, mispairing between homoeologous chromosomes leads to exchanges. HEs can generate homoeologous reciprocal translocations (HRT) or homoeologous non-reciprocal translocations (HNRT), leading to deletions, duplications, and translocations, even when small regions of duplicated DNA are included [96]. Recurrent polyploidy events gave rise to primary and secondary homoeology: homoeology between subgenomes (primary, resulting from a recent event) and homoeology within each subgenome (secondary, arising from older events). Good examples of this are genomes A, B, and C in *Brassica* [124].

Duplications, deletions, and rearrangements can be caused by non-homologous recombination events. Although these abnormalities supposedly occur in almost all evolutionary lineages, a rearrangement is more likely to be fatal in a diploid lineage if a large deletion or duplication is involved [125]. Nonetheless, in polyploids, an extra set of chromosomes facilitates chromosome exchange because when two or more copies of a gene (or genomic region) are present, rearrangements can occur without affecting gamete viability and fertility. In fact, the success of polyploidy in many lineages is partially due to genomic redundancy [126], although it can slow down the respective loss or fixation of deleterious and beneficial alleles [127,128].

HEs and other karyotypic variations have been associated with phenotypic changes in many polyploids [129]. HNRTs and deletions have been correlated with qualitative changes in the expression of specific homoeologous genes and anonymous cDNA amplified fragment length polymorphisms and with phenotypic variation among polyploids Exchanges among homoeologous chromosomes are a major mechanism for creating novel allele combinations and phenotypic variation in newly formed polyploids, generating extensive genetic diversity in a short period of time [15,122,130,131]. Therefore, HEs, duplications/deletions, and chromosome rearrangements may provide an important evolutionary substrate in neoallopolyploids for divergence, speciation, and adaptation [96].

## 9. Neoallopolyploids

In contrast to established allopolyploids, neoallopolyploids have a higher number of synaptic multivalents persisting to MI, resulting in high rates of homoeologous recombination, chromosomal rearrangements, and aneuploidy [15,123,132]. In neoallopolyploids, rearrangements are often accumulated and passed on to subsequent generations. However, since fertility is usually lower, they are selected against during the establishment of a new polyploid species [122,123].

Progenies of hybridization events have variable chromosome constitutions, and this may expand genetic variation and contribute to the success of neoallopolyploids. Variations in chromosome constitution in neoallopolyploids are not random. They are usually genetically balanced, i.e., the lack of one or a pair of chromosomes is compensated by an increased dose of its homoeolog, or, where translocations occur, by equivalent segments of homoeologs. According to Oleszczuk and Lukaszewski (2014) [133], these changes do not alter gene dosages; in other words, new allopolyploids do not suffer any immediate impairment due to random numerical aneuploidy and may benefit from altered dosages of homoeoalleles.

When homoeologs pair, the pattern of chromosome segregation is usually altered: univalents deliver one sister chromatid and paired homoeologs deliver both sister chromatids to each pole. This behavior results in nullisomic gametes for one homoeolog and disomic for the other. Then, during fertilization, when these gametes fuse, either a nulli-tetrasomic or a monotrisomic will be formed, both with compensating chromosome constitutions [134]. On the other hand, the segregation of a bivalent delivers both sister chromatids from a homoeolog to the same pole. Since the formation of bivalents depends on homoeologous CO and chiasmata, at least one of the two sister chromatids is recombined in each chromosome and consists of segments from both homoeologs, and therefore, the gametes should be genetically complete and hence viable [135].

Meiotic restitution is another event that often occurs in neoallopolyploids. It is genetically controlled, at least in wheat [136]. For instance, in wide hybrids, when specific homologous pairs are present, restitution occurs in meiocytes where homologous chromosomes failed to pair [137]. Deviations from normal chromosome behavior during meiotic restitution, such as infrequent homoeologous pairing or early migration of univalents to the poles, are capable of generating unusual chromosome constitutions [135,138] and may explain some linked loci loss patterns [139,140].

Patterns of chromosome segregation may create diverse chromosome constitutions, including deviations in chromosome numbers among the progeny due to unreduced gametes. These deviations boost genetic variation among newly created genotypes, allowing natural selection to favor best fit combinations. Slight alterations in the patterns of truncated meiosis common in hybrids expand the available chromosome compositions amongst the progeny, usually in a genetically balanced way, which can considerably boost the fitness of spontaneously generated allopolyploids [133].

## 10. Genetic Regulatory Systems in Allopolyploids

Over 70% of angiosperms are polyploids, mostly allopolyploids, including wheat, canola, oats, cotton, tobacco, and oilseed rape, which are some of the world’s most important crops. The significant levels of allopolyploidy suggest that species already had mechanisms for sorting homologous and homoeologous chromosomes, leading to allopolyploid fertility [141]. Mechanisms regulating chromosomal pairing were identified in several allopolyploid species. A key study by Sears (1976) [94] pointed out that homoeologous pairing at MI is suppressed by the *Ph1* locus, increasing karyotypic stability and acting as a pairing regulator in wheat. Similar evidence has been reported for *Avena sativa* [142], *Festuca arundinacea* [143], *B. napus* [144], and *Oryza sativa* [145]. Moreover, a relevant indicator for the presence of pairing control genes (PCG) is the mendelian segregation of polymorphic meiotic behaviors, e.g., in *B. napus* [144], *Lolium perenne* [146,147], and *Festuca pratensis* [148].

When PCGs are present in wheat [114,149] and *Lolium* hybrids [150], the proportion of multivalents at zygotene is lower, indicating that the PCGs affect the assembly of initial synapsis and correct SCs among homoeologs, and that more than one gene contributes to allopolyploid diploidization. Indeed, some chromosomes implicated in homoeologous pairing regulation have been identified in *Avena*, *Lolium*, *Festuca*, and *Brassica* [96].

Two important hypotheses have been put forward regarding the origin and evolution of pairing regulators in allopolyploids: (*i*) Alleles suppressing homoeologous recombination already existed in diploid progenitors at low frequencies. These pairing control alleles could be transmitted to the new allopolyploid, enhancing homologous bivalent pairing and fertility [151]. (*ii*) At the time of allopolyploid formation, mutations suppressing homoeologous pairing could have arisen [93]. So far, none of these hypotheses has been supported by direct evidence, and we may also hypothesize that pairing control systems evolved from a combination of evolutionary scenarios, leading to cumulative suppression of homoeologous pairing [152]. Herein, we will discuss genetic meiotic control in two allopolyploid species in which it has been thoroughly described.

## 11. Wheat

Domesticated wheat (*Triticum aestivum*) is an allohexaploid, originated 500,000 years ago from the hybridization of two diploid species, *T. urartu* (AA) and an unknown *Aegilops* species (BB), generating a tetraploid wheat (*T. dicoccoides*; 2*n* = 4*x* = 24; AABB), followed by domestication into *T. dicoccum* and *T. turgidum*. Later on, 10,000 years ago, *T. turgidum* hybridized with *A. tauschii* (DD), forming the allohexaploid wheat (2*n* = 6*x* = 42; AABBDD; see [153]). Each of the seven homologous chromosome pairs has a corresponding homoeolog within the other two genomes, with similar gene order and content [141]. Wheat behaves as a diploid, with every chromosome synapsing and recombining only with its true homolog (for example, 1A pairs only with 1A, but not with 1B or 1D). *Pairing homoeologous 1* (*Ph1*), a dominant locus on the long arm of chromosome 5B identified by Riley et al. (1958) [154], and Sears et al. (1958) [155], is primarily responsible for this phenotypic behavior. Rather than being passed down from a diploid ancestor, the *Ph1* locus is most likely to have originated during the polyploidization process. It was first discovered by scoring the MI phenotype of hexaploid wheat hybrids lacking the 5B whole chromosome, and this kind of deletion mutant was found to control correct pairing in wheat and its hybrids [149,156], and it is assumed to inhibit homoeologous recombination [157].

Wheat × rye interspecific hybridization produces a hybrid consisting of haploid complements from both species. In the Chinese Spring cultivar (CS *ph1b*, Sears, 1976 [94]), a *Ph1* deletion mutant has been widely used in breeding programs. *Ph1* inhibits introgression by suppressing COs between homoeologous chromosomes. Nonetheless, the number of COs rises in *Ph1* mutant wheat–rye hybrids. Further deletion mutants of the *Ph1* locus were subsequently created and studied [158,159,160,161].

## 12. The *Ph1* Locus

Combined cereal synteny and wheat BAC (Bacterial Artificial Chromosome) contiging, with MI analysis of mutants carrying deletions of chromosome 5B, have been used for molecular characterization of the *Ph1* locus [159,160,162]. However, smaller deletions on 5B were characterized later, and the locus was found to be located in a 2.5 Mb region containing two *Ph1* candidate genes, namely *cdc2* [162] and *C-Ph1* [163].

According to Griffiths et al. (2006) [162], the *Ph1* locus is a region containing a cluster of *Cdk2-like* and *S-adenosyl methionine-dependent methyltransferase* (SAM-MTases) genes and a duplicated segment of heterochromatin from chromosome 3B. This heterochromatin segment was found to contain a gene formerly designated as *hypothetical 3* (*Hyp3*, UniProtKBQ2L3T5), which has been reannotated as *TaZIP4-B2* (UniProtKBQ2L3T5) [141,160,162]. Although the *ZIP4* copy on the 5B locus is dominant, the ancestral homoeologous *ZIP4* copies on 3A, 3B, and 3D are still expressed [162,164].

A more detailed BAC library analysis by Al-Kaff et al. (2008) [160] showed that the *Cdk-like* locus on chromosome 5 differs from the locus on chromosome 3 insofar as it harbors a segment of subtelomeric heterochromatin. Furthermore, the Cdk-like cluster on 5B is different than the clusters on 5A and 5D, and sequencing analysis revealed that tandem duplication events gave rise to these loci. Expression studies showed that the Cdk-like locus on chromosome 5B is dominant in transcription control over the analogous Cdk-like loci on chromosomes 5A and 5D, rendering the *Ph1* phenotype specific to 5B. Nonetheless, overall transcription levels are not affected by the deletion of the Cdk-like locus on 5B, since the genes located on chromosome 5A and 5D can offset the transcription levels.

The *Ph1* locus stabilizes polyploidy in wheat by controlling the accuracy of homologous synapsis and regulating CO formation [158]. Early in meiosis, *Ph1* promotes synapsis between homologous chromosomes. In wheat, chromosomes assume a telomere bouquet arrangement where homologs and homoeologs are sorted, independently of *Ph1* [158]. During the telomere bouquet stage, synapsis can occur only between homologous chromosomes in hexaploid wheat. However, in the absence of *Ph1*, homologous synapsis is less efficient, with more overall synapsis occurring after the telomere bouquet has dispersed, when homoeologous synapsis can occur. This non-specific synapsis between homoeologs leads to the low level of multivalents and univalents observed at MI in wheat lacking *Ph1*, indicating that, during the telomere bouquet stage, meiocytes from wheat and wheat–rye hybrids, with and without *Ph1*, exhibit significant differences in the level of synapsis and chromatin structure, implying that homoeologous synapsis is independent of *Ph1* [141,158,165]. Importantly, this observation led to the idea that *Ph1* may promote homologous synapsis rather than preventing homoeologous synapsis.

*Ph1*’s influence on synapsis is mostly likely due to a change in chromatin structure produced by the Cdk-like and SAM-MTase cluster [166]. Cdk2 has been found to play a role in histone H1 phosphorylation, replication, chromatin condensation, and homoeolog synapsis [167,168]. Cdk2 kinase is essential for meiosis and phosphorylates a variety of targets [169,170]. During early meiosis, it co-localizes with mismatch repair proteins to form recombination nodules and telomere regions [171]. Through histone phosphorylation and chromatin remodeling, Cdk2 has been implicated in licensing replication origins [172]. Additionally, in both the presence and absence of *Ph1*, increased histone H1 CDK2-dependent phosphorylation is related to the effect of *Ph1* on synapsis during CO [173]. Altered phosphorylation affects chromatin structure and delays pre-meiotic replication, impacting homologous synapsis and thus allowing homoeologous synapsis to occur [141,173]. In *Arabidopsis* lines carrying mutations in *Ph1CDK2*-like homologs also show reduced synapsis under particular circumstances, implicating these genes in efficient synapsis [174]. In addition, treatment with okadaic acid, a phosphatase activity inhibitor, enhances Cdk2-type phosphorylation and phenocopies the *ph1b* allele by inducing COs [171].

A second effect of *Ph1* occurs later in meiosis, affecting CO formation levels and the progression of MLH1 sites to COs, according to immunolocalization analysis using the MLH1 DNA mismatch repair protein [158]. MLH1 is necessary to resolve DHJ into COs and is part of the primary class I CO route in plants [175]; it is required to resolve DHJ as COs. All MLH1 sites on synapsed chromosomes become COs in plants [176,177]. However, whether *Ph1* is present or not, similar numbers of MLH1 sites are detected in wheat–rye hybrids. The presence of *Ph1* inhibits MLH1 sites from progressing to COs by inhibiting recombination [158]. On the other hand, its absence allows one-third of MLH1 sites to proceed to COs, indicating that it plays a role in homoeologous MLH1 site resolution. In hexaploid wheat, similar numbers of MLH1 sites are found on synapsed chromosomes at diplotene, but only when *Ph1* is present and the number of COs matches the number of MLH1 sites. As a result, the CO level in *Ph1*-deficient wheat and its hybrids is lower than expected.

## 13. The *ZIP4* Gene

In both *Arabidopsis* and rice, *ZIP4* has been shown to have a major effect on homologous COs, but not on synapsis, in contrast to *Ph1* [178,179]. Knockouts of this gene in diploids usually result in sterility, as the elimination of homologous COs leads to pairing failure and incorrect segregation at late MI. Thus, it seems more likely that *ZIP4* is involved in the way *Ph1* influences CO formation. Increased *ZIP4* gene dosage may bias recombination toward homologs rather than homoeologs [166].

According to bioinformatics studies, in certain polyploid plant lineages meiotic recombination genes are the fastest to return to the single copy state, which is thought to be a rapid response for adapting meiotic recombination post whole-genome duplication [180,181,182]. This is the opposite of the effect of *ZIP4*, which has a novel dominant copy. Therefore, the stabilization process after the polyploidization of wheat is assumed to be involved in rapid changes in the content and expression of the genes in homoeologs. This process would facilitate the correct pairing and synapsis of homoeologs.

The evolution of *Ph1* during *Triticum* polyploidization likely explains why wheat has maintained a similar gene content and balanced expression of its homoeologous groups. It is still unclear how meiosis has adapted to cope with allopolyploidy in other plants. However, it has been hypothesized that a reduction in the copy number of meiotic genes (MG) may stabilize the meiotic process after polyploidization [181,183], although in wheat, the presence of *Ph1* is more likely to have enabled the retention of multiple copies of MGs as a strategy to ensure correct chromosome segregation [164]. The discovery of the *TaZIP4* gene as a candidate for the way the *Ph1* locus affects recombination suggests that *TaZIP4* is more involved in meiosis than originally suspected from studies with model systems [178,179].

Finally, it has been suggested that *ZIP4* acts as a scaffold protein containing tetratricopeptide repeats (TPRs), facilitating the assembly of protein complexes and promoting homologous COs [178,179,184].

A co-expression gene network comparative analysis of meiosis-specific genes has shown that three *TaZIP4* homoeologs, 3A, 3B, and 3D (TaZIP4-A1, TaZIP4-B1, and TaZIP4-D1) formed a cluster and were connected to many orthologs of MGs with different functions [164]. The *TaZIP4* copy on 5B (*TaZIP4*-B2), responsible for the *Ph1* phenotype, did not cluster with the *ZIP4* from the group 3 chromosome, given its different expression profile compared to the other homoeologs and its expression in most tissues [141,165,185].

The stabilizing effects of the meiotic gene *TaZIP4*-B2 were explored by Alabdullah et al. (2021) [186]. The removal of *TaZIP4*-B2 via CRISPR resulted in 56% of meiocytes exhibiting meiotic irregularities at MI, chromosome mis-segregation at AI, and 50% of tetrads with micronuclei. A hexaploid wheat mutant (*Ph1b*) with a 59.3 Mb deletion covering *TaZIP4-B2* shows a comparable amount of disruption, with 56% of meiocytes displaying meiotic irregularities. Given the existence of three additional *ZIP4* copies in the wheat genome, the emergence of a meiotic pairing and CO phenotype resulting in decreased fertility with loss of a single copy of *ZIP4* was unexpected. The TaZIP4-B2 copy enhances homologous pairing, synapsis, and CO, whilst repressing homoeologous COs. As a result of the TPR difference between *TaZIP4-B2* and *TaZIP4-B1*, the hexaploid wheat *TaZIP4-B2* phenotypes are most likely the effect of a reduction in the normal functions of group 3 *ZIP4s* (TaZIP4-A1, TaZIP4-B1 and TaZIP4-D1). TaZIP4-*B2* is expected to compete with group 3 *ZIP4s* for loading into meiotic chromosomes due to its early and three-fold higher expression compared to group 3.

The elongation of the chromosomal axis during meiosis [156] and the interaction of the *ZIP4* protein on these axes producing “pairing bridges” between homologs are believed to be involved in *TaZIP4-B2’s* facilitation of homologous pairing. Homolog alignment and pairing are delayed during early meiosis if the degree of homolog elongation differs [187]. The cohesion protein REC8 is needed for proper meiotic chromosome conformation as well as chromosomal axis elongation via the ASY1 assembly [188,189]. *ZIP4* is found near the end of chromatin regions linked with REC8 [178]. The simplest explanation for *TaZIP4-B2’s* capacity to promote homologous pairing is that it reduces homolog elongation, resulting in more comparable conformations and permitting fast attachment of ZIP4 loci, thereby lowering the likelihood of homoeologous pairing later in meiosis [141,156,158].

The interaction between *ZIP4* copies on chromosome 5B and on chromosomal group 3 is believed to be the reason why *TaZIP4-B2* inhibits homoeologous COs. As in other species, group 3 ZIP4s are predicted to handle 85% of homologous COs [178,179]. Given the number of COs found in wheat haploids missing *TaZIP4-B2* [190], they are also assumed to process homoeologous CO activity. The divergent *TaZIP4-B2* copy, on the other hand, exhibits some homologous CO activity but no homoeologous CO activity [185]. As a result, the presence of *TaZIP4-B2* with wheat group 3 *ZIP4s* in chromosomal foci that assemble CO proteins, including MLH1, indicates that only homologous COs, not homoeologous COs, are effectively processed [158,165,166].

*TaZIP4-B2* deletion decreases homologous COs, which leads to an increase in meiotic irregularities at MI [185]. This shows that *TaZIP4-B2* enhances homologous COs and implies that *ZIP4’s* impact on homologous COs might be dose-dependent.

## 14. *Ph2*

The *Ph2* locus was attributed to chromosome 3D by Mello-Sampayo (1968, 1971) see [191,192] who reported multivalent formations at MI in the absence of chromosome 3D in pentaploid hybrids between *T. aestivum* and *T. durum* and *T. aestivum* and *Aegilops*. Since then, two *Ph2* mutants have been discovered: an X-ray induced mutant with a substantial deletion [193] and the EMS-induced mutant *ph2b* [194]. The *Ph2* phenotype was investigated using both mutants, and the locus was narrowed down to 80 Mb located on the terminal portion of the short arm of 3D (3DS), according to the synteny in the wheat and rice regions analyzed [195]. Svačina et al. (2020) [97] reported that this loss encompasses roughly 125 Mb of the short arm of chromosome 3D. The identification of a number of potential meiotic genes on 3DS has emerged from research aimed at identifying *Ph2*. These genes include WM1 [196,197], WM3 [198], WM5 [199], and TaMSH7 [200,201]. Despite these efforts, the region is too large to draw conclusions regarding the *Ph2* causal sequence. The EMS-induced *Ph2b* mutant [194], which has a point mutation at the *Ph2* locus, provides some hope for finding the candidate sequence [202].

The *Ph2* locus differs from *Ph1* insofar as it has less influence on homolog pairing in wheat [203,204]. Both Martinez et al. (2001) [204] and Sánchez-Morán et al. (2001) [205] noticed no discernible effect on homoeologous chiasmata in the presence of *Ph1* and absence of *Ph2*, with the exception of an increase in univalents. Sears (1977, 1982) [193,206] had previously demonstrated that in wheat and closely similar species hybrids, a moderate number of homoeologous chiasmata occurred in the absence of *Ph2* and presence of *Ph1*. In the case of wheat–rye hybrids lacking the *Ph2* locus, Prieto et al. (2005) [207] also found a moderate amount of homoeologous chiasmata. Nevertheless, chromosomal associations exclusively occur between wheat chromosomes, whereas chromosome associations in wheat–rye hybrids are infrequent. When homologs are present, *Ph2* has a more limited functional role; however, it may inhibit connections between homoeologs in the absence of homologs. Furthermore, *Ph2* is not involved in the identification of homologs but instead impacts the development of synapsis [204,207]. As highlighted by Boden et al. [208], interaction between *Ph1* and *Ph2* should not be overlooked.

Comparative genetics studies were performed by Sutton et al. (2003) [195], to investigate the potential genes implicated in the *Ph2* phenotype. However, no candidate responsible for a mutant phenotype equivalent to the *ph2a* was found. Single nucleotide polymorphism (SNP) based genotyping and exome analysis with the goal of accurately delineating the *ph2a* deletion breakpoint were performed. The *Ph2* locus was found to be within a 14.3 Mb genomic gap, and 24 genes were discovered within the deleted region. The gene TraesCS3D02G119400, coding for a DNA mismatch repair protein (TaMSH7-3D), was found in the 14.3 Mb interval. It has 17 exons and 16 introns with a total length of 9747 bp [202].

On the basis of RNA-seq data analysis, TaMSH7-3D is expressed in anthers at PI. Together with TaMSH7-3A and 3B homoelogs, TaMSH7-3D is expressed throughout PI, lending support to a function for TaMSH7-3D in homoeologous recombination regulation [164]. TaMSH7 (*MutS* homolog 7) is a DNA mismatch repair (MMR) family member found only in plants. These highly conserved proteins are critical for genome integrity because they constitute the first stage of the MMR pathway [209]. In a hexaploid wheat x *Aegilops variabilis* hybrid, the lack of functioning TaMSH7-3D causes a 5.5-fold increase in CO frequency and is thought to play a role in recombination partner selection (homologous vs. homoeologous) by increasing the instability of homoeologous recombination. MMR proteins have been shown to play a role in detecting mismatches in heteroduplex DNA (after DNA strand exchange) and encouraging the dissociation of invading strand DNA, which is a process known as heteroduplex rejection [210]. MSH7 may also play a role in limiting ectopic recombination, which causes highly deleterious chromosomal rearrangements in diploid species and could potentially provide an immediate advantage to newly formed allopolyploids by ensuring meiotic stability and, as a result, fertility in these novel allopolyploids. The discovery of TaZIP4-B2 and TaMSH7-3D, the two major genes governing homoeologous recombination in bread wheat, opened up the possibility of understanding how they act and interact. TaZIP4-B2 promotes homologous bivalent formation by preventing recombination between homoeologous chromosomes generated by COs, according to new research from the ‘G. Moore group’ [141,158]. TaMSH7-3D and TaZIP4-B2 may operate sequentially with distinct modes of action, implying that homoeologous recombination in polyploid bread wheat is a multilayered mechanism. Sears (1976) [94] revealed that *Ph1* is twice as strong as *Ph2*, and that these changes have an additive impact in increasing homoeologous recombination, as seen in wheat x *Aegilops* hybrids [211]. Thus, combining TaZIP4-B2 and TaMSH7-3D mutations may provide a way of increasing the effectiveness and simplicity of introducing wild related chromosomal regions into wheat, allowing for the production of genetically distinct, attractive wheat cultivars [202].

## 15. *Brassica* and the Prevalence of Bivalent Pairing

Brassiceae is one of the most morphologically distinct tribes within the Brassicaceae family (Cruciferae). It is a monophyletic group of species that has undergone whole-genome triplication. Extensive chromosome rearrangements, including fusions and/or fissions, resulted in chromosome number variation for the three diploid *Brassica* species, *B. nigra* (BB; *2n* = 16), *B. oleracea* (CC; *2n* = 18), and *B. rapa* (AA; *2n* = 20) [212]. Subsequent spontaneous hybridization between the ancestors of these three diploid species, followed by chromosome doubling [213], introduced an additional layer of duplication within the genomes of the three allotetraploids, *B. juncea* (AABB; *2n* = 36), *B. napus* (AACC; *2n* = 38), and *B. carinata* (BBCC; *2n* = 34). Alignment of the A- and C-genomes of *B. napus* allowed the identification of regions with primary homoeology (i.e., regions from the A and C genomes that share a recent common ancestry) [214].

Natural euploid *B. napus* (AACC, *2n* = 38) exhibits predominantly 19 bivalents at MI, with a preference for homologous chromosome pairing and disomic inheritance. In the resynthesized *B. napus*, preferential CO formation between homologs was reported as an immediate response to polyploidization and a clear predominance of bivalent formation, with 80–85% of pollen mother cells (PMC) exhibiting 19 bivalents [215,216].

However, not all resynthesized *B. napus* bivalents are formed between homologs. Some allosyndetic bivalents between A and C homoeologs were observed [214], and these bivalents are formed in two ways: through regions of intra- or intergenomic homology, which resulted from whole-genome duplications in the common ancestor of *B. rapa* and *B. oleracea* [124,212]; or through homoeologs carrying segmental duplications that occurred after the polyploidy events [217,218]. Univalents and multivalents were also observed in resynthesized plants, confirming that meiotic behavior was not fully diploidized. Therefore, in comparison to natural *B. napus*, the irregular meiosis of resynthesized *B. napus* generates a higher proportion of homoeologous exchanges resulting in HNRTs [219,220].

Notably, Jenczewski et al. (2013) [119] showed that the distribution of the number of univalents among haploids was consistent with the segregation of a biallelic gene, *Pairing regulator in B. napus* (*PrBn*), against a background of polygenic variation. This study reported a high level (75%) of two to three bivalents in the respective haploid varieties Darmor-bzh and Yudal, resulting from both auto- and allosyndesis within and between the A and C genomes of oilseed rape. In *B. oleracea* and *B. rapa*, the pairing of two homoeologs originating from the same genome (autosyndesis) has been reported [221] as a result of intragenomic duplications [222,223]. Previously, high-pairing haploids of oilseed rape exhibited meiotic behavior similar to that of hybrids between *B. rapa* × *B. oleracea* [216]. In other words, these haploids provide evidence that the differences between the high- and low-pairing haploids are genetically controlled [145].

## 16. *PrBn* Molecular Characterization and Function

New insights into the genetic architecture of *PrBn* showed that the hereditary components of homoeolog pairing are polygenic [119,224]. Using molecular markers (RAPD and AFLP), one linkage group of ≈70 cM was identified in which the *PrBn* locus was mapped (10–20 cM interval) on a linkage group designated DY15 attributed to chromosome C9. In addition, three to six minor quantitative trait loci (QTL) on C1 and C6 had minor additive effects on the number of univalents but do not seem to have interacted with *PrBn*. A further two to three loci that interact epistatically with *PrBn* were also detected.

In *B. napus*, recurrent polyploidy has driven extensive variation in the determinants of CO suppression between homoeologs. The natural variation in meiotic behavior among *B. napus* allohaploids is consistent with the segregation of two *PrBn* alleles, which is the expected composition resulting from a *B. napus* double origin [119].

The current understanding of CO variation in *B. napus* is based on cytological observations at MI and on genetic surveys of intergenomic exchanges in *B. napus* allohaploid progenies. Nicolas et al. (2009) [225] discovered that the *PrBn* locus (and the genes it interacts with) control the frequency, but not the distribution of COs between homoeologs in *B. napus* haploids, and between homologs during meiosis of triploid ArAnC (Ar = *B. rapa*; An = *B. napus*) hybrid plants. The threefold difference in the number of COs formed between homoeologs is the cause of the meiotic behaviors observed in Darmor-bzh (high-pairing) and Yudal (low-pairing) haploids. Given that the action of *PrBn* and the genes with which it interacts genetically determine these two meiotic phenotypes, it was concluded that these loci influence recombination between homoeologs. However, *PrBn* does not affect the level of homologous recombination in tetraploid ArAnCC hybrids, suggesting that its effect on recombination depends on the background karyotype.

Although the exact origin of the karyotypic influence on CO variation is uncertain, at least two ideas have been put forward. Firstly, it was proposed that there is a *PrBn* dosage effect on the *B. napus* C genome. One copy of the gene(s) carried by the Yudal C genome would lead to fewer COs than one copy of the gene(s) carried by the Darmor-bzh C genome, but two copies of gene(s) carried by the C genomes would provide the same number of COs [144,225,226,227]. Dosage effects have been shown to be common among the genes regulating chromosome pairing and recombination in polyploids [44,96], including *Brassica* [228,229]. Secondly, the presence of chromosomes that remain unpaired during meiosis (in haploids and triploids, but less frequently in tetraploids) may trigger genotype-dependent changes in the progression/completion of meiotic steps [230,231].

Synthetic *Brassica* allohexaploids [232] and allotetraploids [233] have long been known to be meiotically unstable, and synthetic *B. napus* is often extremely unstable, putatively due to the close relationship between the A and C genomes [15,123,131]. In contrast, natural allotetraploid species *B. juncea*, *B. carinata*, and *B. napus* are fully stable and fertile. More recently, Tian et al. (2010) [234] produced *B. rapa* × *B. carinata* allohexaploids exhibiting increased fertility and percentages of offspring with 2*n* = 54 up to the 4th generation using different genotype combinations. Zhou et al. (2016) [235] found high fertility and stable breeding behavior in allohexaploids from *B. rapa* × *B. carinata* and *B. juncea* × *B. oleracea*, and lower fertility in allohexaploids from newly combined diploid genomes.

Fertility and meiotic stability in novel *Brassica* allohexaploids have been investigated to determine which factor could influence these traits, and studies on homozygous (A2) and heterozygous (H2) allohexaploids have shown a variation in fertility traits and meiotic configuration. For example, A2 displays low pollen fertility and a high level of chromosome loss, whereas in H2, high pollen fertility and an average of 49 chromosomes were found [236]. The direction of unbalanced homoeologous exchanges (which subgenome was lost or duplicated, which is a potential mechanism for biased fractionation), the loss or presence of univalent chromosomes, and inheritance of particular genomic regions from the allotetraploid parents have all been identified as major factors influencing the fertility and meiotic stability of novel allohexaploid hybrids. Replacing an A-genome fragment with a C-genome fragment was found to compromise fertility. In allohexaploid hybrids and synthetic *B. napus*, bias in the directionality of translocations is a driving force for genome size reduction and biased fractionation, whereby gene copies from one subgenome are preferentially lost [237]. Interestingly, it has been proposed that a subgenome with a higher number of transposable elements is more likely to be lost in allopolyploids as a result of biased genome fractionation [238,239,240,241]. A preferential loss of the larger, transposable-element-rich C genome originated in *B. oleracea* has been shown to occur over evolutionary time [238,242,243].

## 17. *BnaPh1*

Higgins et al. (2021) [14] were the first authors to describe the *BnaPh1* locus. Established and resynthesized *B. napus* lines were compared to search for possible QTLs that may influence mispairing and subsequent homoeolog recombination in a segregating doubled-haploid (SGDH) population. The quantification of recombination events on the homoeologs allowed putative meiosis-specific genes to be identified.

A *B. napus* SGDH was used by Clarke et al. (2016) [244] to generate a genomic map with 21,000 SNPs, and the *BnaPh1* (*B. napus Pairing homoeologous 1*) locus was mapped on chromosome *BnaA9*. Depending on the dataset, this QTL explained 32–58% of the overall variance, and two minor QTLs were positioned on the *BnaA3* and *BnaC7* chromosomes. With a length of 12.8 Mb, the *BnaA9* QTL comprises the centromeric region and, as expected, it exhibited low homologous CO rates and significant linkage disequilibrium.

Both *A. thaliana* and *B. napus* are cruciferous species. On the basis of those QTL locations, *A. thaliana* meiosis-related genes were investigated to search for orthologs in the *B. napus* genome. Reciprocal exchange, deletion/duplication, and synaptic partner switch QTLs on *BnaA3*, *BnaA9*, and *BnaC7* were found to have 12 candidate genes.

RPA1C (Replication Protein A 1C) and MUS81 (MMS and UV Sensitive 81) are two of the five genes found to be associated with the *BnaA9* QTL. RPA1C acts in double-strand break repair at early meiosis in *A. thaliana* [245]. A further DNA repair protein implicated in the interference-free CO route is the endonuclease MUS81 [246,247]. Researchers had previously exploited the *BnaC9* copies of MUS81 and RPA1C in regard to the *PrBn* locus but found no significant differences in their expression between the respective high and low homoeologous pairing lines, leading them to conclude that neither gene was responsible for the *PrBn* phenotype [248]. However, because the *BnaPh1* locus was mapped in allotetraploids rather than allohaploids, and because levels of meiotic transcription in wheat have been shown to be stable in the presence and absence of the *Ph1* locus [165], either MUS81 or RPA1C, or possibly an unidentified gene, could be responsible for the QTL discovered.

Researchers found two other smaller QTLs harboring four and three meiotic genes, respectively, one on *BnaA3* and the other in the homologous region on *BnaC7*. MSH3 is one of the genes found on minor QTLs, a homolog of the *MutS* gene, which controls mismatch repair in *Escherichia coli* [249]. In *A. thaliana*, six *MutS* homologs are present. MSH4 and MSH5 have recognized functions in meiotic recombination [250,251], whereas MSH2, MSH3, MSH6, and MSH7 are crucial for DNA repair in *A. thaliana* [252]. MSH2 on chromosome C3 was one of the potential meiotic instability loci discovered by Gaebelein et al. (2018) [232] in *B. napus*, whereas MSH7 has also been identified as a potential *Ph2* gene in wheat [200,201,202].

Gonzalo et al. (2019) [183] found that homologous recombination in *B. napus* allohaploids decreased when MSH4 was reduced to one functional copy, while homologous recombination in allotetraploids remained unaffected. Due to the low incidence of naturally occurring homoeologous recombination in *B. napus*, the effect of decreasing MSH4 on the rate of homoeologous recombination in allotetraploids could not be established. This indicates either that *BnaPh1* is haplo-insufficient or that it does not specifically counter the formation of early recombination intermediates and COs between homoeologs. Therefore, *BnaPh1* is thought to help promote the maturation of recombination intermediates between the two homologs in the euploid lines, but it does not prevent COs between homoeologs in haploid lines [253]. This research suggests that lowering the number of functional gene copies for meiotic genes might be an essential evolutionary adaptation for polyploid meiotic stability. The *PrBn* QTL was mapped to chromosome *BnaC9* in segregating allohaploid lines [224]; interestingly, the *BnaC9* and *BnaA9* loci are located in a homoeologous region. The *BnaPh1* locus in the diploid *B. rapa* was explored to determine if genes present in the presumed progenitor *B. napus* had been deleted; however, there was no evidence of missing meiosis-related genes for this region [14]. As a result, the presence of meiotic controlling genes in homeologous regions could be attributed to the process of polyploidization, which results in the generation of paralogous genes. However, further research on both QTLs is needed to support this hypothesis.

## 18. Meiotic Proteins and Crossover Formation

Many proteins that play a role in CO formation have been identified [158,179,208,254]. Since the study conducted by Riley and Chapman [154] how meiosis has adapted to deal with allopolyploidy has been deciphered only in wheat. A duplication of the *ZIP4* gene within the *Ph1* locus prevents the maturation of COs between nonhomologous chromosomes [154,158,166,178,179]. The CO Class I or ZMM pathway includes a set of critical proteins in plants, such as MER3, MSH4, MSH5, SHOC1, HEI10, PTD, and ZIP4. The possible impact of genetic regulation on CO formation was illustrated by Grandont et al. (2014) [95], whose findings showed that during PI, the spatial–temporal localization of HEI10 is the same in *B. napus* euploids as in *A. thaliana* and rice [255,256,257]. Thus, the relocation of HEI10 in *B. napus* reflects the progressive formation of recombination intermediates in the ZMM CO pathway. This progression varies among genotypes. For instance, in Yudal, the transition from early to late HEI10 occurs in earlier stages of PI compared to Darmor-bzh.

Both *Brassica* allohaploids (Darmor-bzh and Yudal) form distinct numbers of class I COs, according to the immunolocalization of MLH1 I, suggesting that the progression of early meiotic recombination is essentially the same regardless of whether recombination intermediates are formed between homologs or homoeologs. Only a small fraction of HEI10 and MLH1 foci were found to be colocalized at diakinesis in *B. napus* allohaploids, whereas this was systematically found in euploids. As proposed for haploid *Arabidopsis* [258] and hexaploid wheat [158], ‘stand-alone’ MLH1 foci could mark the locations where COs eventually failed or occurred between sister chromatids. A fraction of late recombination intermediates may still be in the process of resolution at diakinesis in *B. napus* allohaploids, resulting in some HEI10 foci persisting longer than usual on chromosomes without producing the conditions required for MLH1 loading. Separate HEI10 and MLH1 loci may also reflect the aberrant behavior of meiotic proteins, with MLH1 loading and off-loading irrespective of HEI10 [95].

In *Brassica*, there was a reduction in copy number for genes encoding MSH4, MSH5, MER3, and ZIP4 following independent WGDs, although SHOC1 and HEI10 showed higher duplicate retention rates. Higher HEI10 duplicate retention is consistent with the most widely accepted theory that explains the fate of gene duplicates post-WGD. MSH4 is essential to ensure normal CO numbers between homologous chromosomes and is therefore required to ensure fertility. Normal levels of homologous CO mitigate against MSH4 gene duplicate loss; thus, CO formation between homologous chromosomes fluctuates in a dosage-sensitive manner. CO formation is at its maximum when all MSH4 copies are functional, and it gradually decreases with the number of copies, approximating zero when all MSH4 copies are non-functional [183].

Therefore, the modulation of the entire ZMM pathway, or at least part of it, could contribute to meiotic stabilization in allopolyploids. It is unclear whether MSH4 and ZIP4 act on the same step of the ZMM pathway, or even whether their specific roles are conserved between species.

## 19. The Consequences of Meiosis for Genetic Mapping in Auto- and Allopolyploids

The use of genetic analysis in polyploids can be traced back to the work of Muller (1914) [259], who investigated data on the tetraploid *Primula sinensis* previously published by Gregory (1914) [260] and proposed the first polysomic segregation model. In the first half of the twentieth century, several authors addressed the complex inheritance patterns and genetic linkage properties in polyploid organisms [261,262,263,264,265,266,267,268,269,270,271,272]. Although these studies offered key insights into polyploid inheritance theory, they were in practice limited to scarce morphological markers. A few traits were studied, such as the color and shape of the stigma [259,260], petal color [261], and style length [266]. In addition to the low availability of these traits, the complexity of their segregation in experimental populations hindered application in real scenarios, which meant that linkage studies on polyploid species lagged behind studies on diploids. This situation eventually changed with the advancement of recombinant DNA technology in the early 1990s. Wu et al. (1992) [273] and Sorrells (1992) [274] proposed the use of single-dose (SD, or simplex) molecular markers based on restriction fragments to assess allelic variation in polyploids. SD markers display genetic polymorphisms in a single parental homologous chromosome (for example, Aaaa vs. aaaa in tetraploids). When present in one parent, the single variation results in a 1:1 segregation ratio. This approach allows standard diploid techniques to be used for linkage analysis and map construction. Since SD-based mapping does not depend on regular polyploid meiosis [274], it is to this day an extremely valuable technique, even after the development of modern genotyping technologies.

In allopolyploids, bivalent pairing between specific pairs of chromosomes will occur most of the time. Thus, there is no essential difference between the analytical linkage procedures for diploids and allopolyploids when constructing genetic maps. On the other hand, as stated earlier, chromosome pairing in autopolyploids is often unpredictable, and complex meiotic configurations can occur [275]. Three different types of autopolyploid chromosomal segregation have been proposed:

1. *Random chromosome segregation* [259], where the gametes are formed by *p/2* homologous chromosomes selected from *p* chromosomes; for example, in an autotetraploid genotype where *p* = 4, the genotype (A_1_, A_2_, A_3_, A_4_) can yield six different balanced gametes (A_1_A_2_, A_1_A_3_, A_1_A_4_, A_2_A_3_, A_2_A_4_, and A_3_A_4_) with same expected proportions of 1/6.
2. *Random chromatid segregation* [262], where the gamete is formed by *p/2* homologous chromosomes selected at random from *2p* possible chromatids due to double reductional segregation; thus, in addition to the heterozygous classes presented in the previous autotetraploid example, four extra homozygotic types are expected (A_1_A_1_, A_2_A_2_, A_3_A_3_, A_4_A_4_), with proportions of 1/7 for heterozygous classes and 1/28 for homozygous classes.
3. *Maximum equational segregation* [264,265]: where a double-reduction coefficient (α is used to regulate the proportion of extreme cases described in items 1 and 2 above.

The number of possible genotypes in a cross between two autopolyploid individuals with random chromosome segregation is given by the square of the number of gametes. Table 1 gives examples of gamete and genotype numbers in a biparental cross for different even ploidy levels. Note that a linear increment in the ploidy level results in a steep increment in the possible genotype numbers.

**Figure 2:**
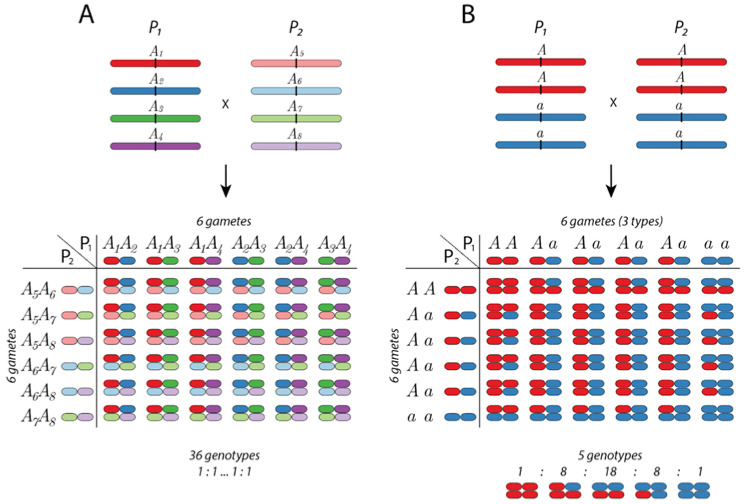
Thirty-six possible genotypes in an autotetraploid cross. (**A**) Completely informative multiallelic scenario, in which all the genotypes formed by the combination of 6 gametes can be differentiated and segregated with equal probability. (**B**) Biallelic scenario, in which both parents have two doses (duplex marker), in which case the genotypes collapse into five different classes segregating in a 1:8:18:8:1 ratio.

In recent years, most polyploid mapping studies have been based on SNPs identified through pre-assembled arrays or genotyping-by-sequencing (GBS) techniques [276,277,278,279,280]. As a result of these techniques and the biallelic quantitative nature of these platforms, the high number of polyploid genotypic classes in Table 1 is expressed in terms of *dosages*. Thus, regardless of the 36 possible genotypic classes in tetraploid biparental populations, they are scored in up to five genotypic classes, i.e., AAAA, AAAa, AAaa, Aaaa, or aaaa. This means that when dosage-based markers are used, the multiple genotypes in a polyploid biparental cross collapse into a lower number of classes if biallelic markers are used (Figure 2).

Several methods and computer programs have been implemented to convert quantitative molecular inputs into binary dosage markers [281,282,283,284]. The corresponding output produces dosage scores for SNPs along the genomes of all individuals in the population, which is used as the starting point for further genetic mapping analysis.

Genetic mapping in polyploid species began with the use of SD molecular markers [273]. As a result, separate maps were generated, one for each homologous chromosome for each parent; i.e., the expected number of linkage groups is the basic chromosome number multiplied by the parent’s ploidy level. A plethora of genetic maps was constructed using this approach in potato [277,285], sugarcane [286,287,288,289,290], sweet potato [291,292], strawberry [293,294], rose [295], and many other species. Some of these studies incorporated multiple-dose (MD) markers into a framework of SD-based maps. However, limitations in the genotyping technology and the complex nature of polyploid inheritance mean that the potential of MD markers cannot be fully exploited in a complete haplotypic inheritance analysis.

The use of multiple-dose markers in polyploid genetic mapping became more widespread with the advent of high-throughput DNA technologies, which allowed thousands of genomic positions to be assessed using SNPs. For a review of genetic analysis in polyploids using SD and MD markers see Bourke et al. (2018) [296]. In the following section, we describe two examples of polyploid maps constructed using the GBS approach. In the first, a sugarcane genetic map was built using exclusively SD markers. The second relates to the autohexaploid sweet potato and involves the use of a variety of dosage-based markers to build a multilocus genetic map. The sweet potato multilocus map facilitated a detailed study of the transmission patterns and meiotic characteristics of this complex hexaploid species.

## 20. Case Studies

### 20.1. Sugarcane

Sugarcane (*Saccharum* spp.) belongs to the Poaceae family and is a highly complex polyploid with recent interspecific hybridization [297]. Cultivated sugarcane was produced by crossing high sugar content species *Saccharum officinarum* (2*n* = 80, *x* = 10) and *S. spontaneum* (2*n* = 40–128, *x* = 8), a wild species with high fiber content [298]. This cross resulted in the so-called *Saccharum* complex with ploidy levels varying from five to 16, often with aneuploidy [297,299,300]. Most of the sugarcane linkage maps to date were produced using SD markers, adding a few duplex and triplex markers [286,287,288,289,290,301]. SD markers are usually the most practical choice for complex polyploid linkage studies because their inheritance is not affected by the ploidy type or level. Although they contain little or no information for assigning homolog chromosomes to homology groups, double SD markers (present in both parents, segregating 3:1 or 1:2:1) can be used to merge the genetic maps of both parents in a biparental population. Garcia et al. (2006) [290] used double SD markers to integrate parental maps for a sugarcane population derived from a cross between two commercial varieties. The authors implemented a joint maximum likelihood method to build maps using simplex and double-simplex markers. The double-simplex markers were used to establish linkages between simplex markers, integrating information from both parents into a joint map.

More recently, the introduction of platforms for genotyping by high-throughput DNA sequencing led to the construction of saturated genetic maps for sugarcane. Balsalobre et al. (2017) [302] used GBS-based and gel-based SSR markers to build a single-dose map, subsequently mapping yield-related quantitative trait loci (QTL). After discovering the SNPs and performing genotype calling, the authors obtained 7678 single-dose high-quality SNPs to build the genetic map. Nine hundred and ninety-three markers were positioned in the final linkage map and distributed over 223 linkage groups clustered in 18 homologous and homoeologous groups (HGs). You et al. [303] used a 100K Affymetrix Axiom Sugarcane SNP array to genotype a full-sib family derived from the Green German and IND81-146 varieties, and a selfing population derived from CP80-1827. The maps obtained revealed higher numbers of markers than previous studies, ranging from 3482 mapped single-dose markers for the Green German parent to 536 for CP80-1827 (see 304 for the comparison).

Although sugarcane genetic maps are becoming denser and covering higher fractions of this complex genome, the lack of high-quality multiple-dose markers precludes any appropriate assignment of homologs to homology groups. Other less complex species, such as potato, rose, blueberry, chrysanthemum, and sweet potato have benefited from multiple markers, resulting in more effective QTL mapping and meiotic studies, as described in the following case study.

### 20.2. Sweet Potato

The hexaploid sweet potato (*Ipomea batatas* (L.) Lam. 2*n* = 6*x* = 90) is an important crop worldwide, serving as a staple food in several developing countries due to its high nutritional value [304]. It is a naturally occurring polyploid with two polyploidization events traced back to 0.8 and 0.5 million years ago [305]. As observed by Gustafsson and Gadd (1965) [306] and Magoon et al. (1970) [307], meiosis in the cultivated sweet potato is regular, with a prevalence of bivalent formations and a constant hexaploid level. Thus, in contrast to sugarcane, the study of inheritance patterns is manageable using appropriate genomic tools and analytical pipelines. In common with sugarcane, the first genetic maps constructed for sweet potatoes were based on simplex markers, incorporating multiple-dose markers into the existing framework map built using simplex markers [291,292,308,309,310,311].

Mollinari et al. (2020) [279] used GBS-based SNP markers to study the inheritance system in a biparental hexaploid sweet potato cross between the Beauregard and Tanzania cultivars. The genomes of two related diploid sweet potatoes were used to anchor *Ipomea trifida* and *I. triloba* SNPs. The authors obtained 30,684 high-quality markers, 60.7% simplex and double-simplex and 39.3% multiplex, combined with a recently developed algorithm to construct multilocus genetic maps in complex polyploids [312]. Due to the abundance of high-quality multiplex markers and novel mapping methods, they assembled an integrated map for both parents and phased homology groups for all parental homologous chromosomes. The resulting map indicated 96.5% and 83.1% collinearity between *I. batatas* and its diploid relatives *I. trifida* and *I. triloba*, respectively. The offspring’s haplotypic composition was inferred in terms of the probability of inheritance of the parental homologous chromosomes, and several meiotic characteristics were investigated. The authors also found that 73.3% of the parents’ meiotic configurations were resolved into bivalents, 15.7% were resolved into multivalent signatures, and 11.0% were inconclusive.

Moreover, the studied population exhibited vastly hexasomic inheritance mechanisms in all linkage groups, providing stable allele transmission. A similar analysis was also conducted on the tetraploid potato. The meiotic configuration estimated using the phased map strongly corroborated the results obtained using cytological techniques [313], which is advantageous for evaluating meiosis by straightforward extension of map construction.

## Figures and Tables

**Table 1:** Number of possible gametes for one locus with no double-reduction and number of possible genotypes generated by their combination given even ploidy levels.

Ploidy Level	Number of Gametes $\left(\begin{array}{c} p\\ \frac{p}{2} \end{array}\right)$	Number of Genotypes ${\left(\begin{array}{c} p\\ \frac{p}{2} \end{array}\right)}^{2}$
2	2	4
4	6	36
6	20	400
8	70	4900
10	252	63,504
12	924	853,776

## Data Availability

Not applicable.
